# Rho Kinases Regulate the Renewal and Neural Differentiation of Embryonic Stem Cells in a Cell Plating Density–Dependent Manner

**DOI:** 10.1371/journal.pone.0009187

**Published:** 2010-02-12

**Authors:** Tzu-Ching Chang, Yen-Chung Chen, Ming-Hua Yang, Chien-Hung Chen, En-Wei Hsing, Bor-Sheng Ko, Jun-Yang Liou, Kenneth K. Wu

**Affiliations:** 1 Institute of Cellular and System Medicine, National Health Research Institutes, Zhunan, Taiwan; 2 National Taiwan University College of Medicine, Taipei, Taiwan; 3 University of Texas Health Science Center, Houston, Texas, United States of America; The Beatson Institute for Cancer Research, Glasgow, United Kingdom

## Abstract

**Background:**

Rho kinases (ROCKs) mediate cell contraction, local adhesion, and cell motility, which are considered to be important in cell differentiation. We postulated that ROCKs are involved in controlling embryonic stem (ES) cell renewal and differentiation.

**Methodology/Principal Findings:**

CCE, a murine ES cell, was treated with Y-27632 for 48 to 96 hours and colony formation was evaluated. Y-27632 blocked CCE colony formation and induced CCE to grow as individual cells, regardless of the initial seeding cell density either at 10^4^/cm^2^ (“high” seeding density) or 2×10^3^/cm^2^ (“low” density). However, at high seeding density, Y-27632–treated cells exhibited reduction of alkaline phosphatase (AP) staining and Oct3/4 expression. They expressed SOX-1, nestin, and MAP2c, but not βIII-tubulin or NG-2. They did not express endoderm or mesoderm lineage markers. After removal of Y-27632, the cells failed to form colonies or regain undifferentiated state. Silencing of ROCK-1 or ROCK-2 with selective small interference RNA induced CCE morphological changes similar to Y-27632. Silencing of ROCK-1 or ROCK-2 individually was sufficient to cause reduction of AP and Oct3/4, and expression of SOX-1, nestin, and MAP2c; and combined silencing of both ROCKs did not augment the effects exerted by individual ROCK siRNA. Y-27632–treated CCE cells seeded at 2×10^3^ or 6.6×10^3^ cells/cm^2^ did not lose renewal factors or express differentiation markers. Furthermore, they were able to form AP-positive colonies after removal of Y-27632 and reseeding. Similar to ROCK inhibition by Y-27632, silencing of ROCK-1 or ROCK-2 in cells seeded at 2×10^3^/cm^2^ did not change renewal factors.

**Conclusions/Significance:**

We conclude that ROCKs promote ES cell colony formation, maintain them at undifferentiated state, and prevent them from neural differentiation at high seeding density. ROCK inhibition represents a new strategy for preparing large numbers of neural progenitor cells.

## Introduction

The mammalian Rho-associated coiled-coil forming protein kinase (ROCK or ROK) comprises ROCK-1 (ROKβ) and ROCK-2 (ROKα) which contain highly conserved amino-terminal and substantially different carboxy-terminal domains [1], [2]. ROCK is a major downstream effector of RhoA GTPase. RhoA binds to the coiled coil region of ROCK and activates ROCK catalytic activity [3]. Activated ROCK mediates actin-myosin contraction, stress fiber formation and local adhesion by targeting downstream kinases and phosphatases resulting in increased myosin light chain phosphorylation [4]. Activated ROCK induces neurite retraction [5] while selective ROCK inhibitor, Y-27632, as well as ROCK dominant negative mutants promote neurite formation [6]. Y-27632 rescues collagen-induced arrest of neurite sprouting and elongation in cultured rat neurons [7]. Recent studies have shown that ROCK is involved in cytokinesis and mitosis. It was proposed that ROCKs are required for contraction of the cleavage furrow [8], and ROCK inhibition was reported to retard cytokinesis and impair cytokinetic segregation of glial filaments [9].

ROCKs may be involved in cell differentiation. It was reported that ROCKs are required for myogenesis from embryonic fibroblasts [10] and for skeletal muscle differentiation and maturation [11], [12]. The RhoA/ROCK signaling pathway was implicated in keratinocyte differentiation [13]. However, little is known about the involvement of ROCK in stem cell differentiation. It was reported that RhoA regulates bone marrow-derived mesenchymal stem cell (BM-MSC) differentiation into adipogenic and osteogenic lineages [14]. Y-27632 was reported to potentiate the effect of CoCl2 on transdifferentiation of BM-MSC into mature neurons, although Y-27632 alone had no effect [15], [16]. To determine whether ROCKs are directly involved in embryonic stem (ES) cell differentiation, we treated a murine ES cell, CCE with Y-27632, H-89 or RNAi and evaluated changes in morphology, renewal factors and differentiation markers. The results show that ROCKs are involved in CCE differentiation in a cell density dependent manner. At a threshold seeding density (10^4^ cells/cm^2^), Y-27632 or selective ROCK-1 or ROCK-2 small interference RNA (siRNA) induced similar morphological changes accompanied by loss of alkaline phosphatase (AP) and Oct3/4 and expression of SOX-1, nestin and MAP2c but not markers of other lineages. At low seeding densities, CCE grew as individual cells and retained AP, Oct3/4, nanog and SOX-2 without increased expression of neural progenitor markers despite Y-27632 or RNAi treatment. Y-27632-treated CCE cells seeded at a low density regained ability to form colonies after removal of Y-27632 whereas those seeded at a high density had undergone irreversible differentiation and were unable to form colonies.

## Materials and Methods

### Cell Culture

CCE, an ES cell derived from 129/Sv mouse strain, was obtained from StemCell Technologies, Inc. with permission from Drs. Robertson and Keller (Vancouver, Canada) [17]. CCE was cultured in Dulbecco's modified Eagle's medium (DMEM) supplemented with 15% fetal bovine serum (FBS) (Hyclone, Logan, UT), 100 U/ml of penicillin, 100 µg/ml of streptomycin, 1 mM sodium pyruvate, 0.1 mM non-essential amino acids, and 10 ng/mL leukemia inhibitory factor at 37°C in a humidified 5% CO_2_ atmosphere.

### Cell Treatment

Trypsinized CCE cells were treated with Y-27632 (10 µM) or vehicle for 1 h prior to seeding at different densities. At various time points after seeding, colony formation and cell morphology were examined under phase-contrast microscopy. In separate experiments, the suspended CCE cells were seeded at different densities for 24 h and Y-27632 was added to the culture dish. Morphological and marker changes were analyzed at 48–96 h. To examine the capability of Y-27632-treated cells to form colonies, Y-27632-treated CCE cells were washed, trypsinized, and reseeded. Colony formation was analyzed with alkaline phosphatase (AP) staining. Vehicle and retinoic acid (RA)-treated cells were included as controls.

### Analysis of Renewal Markers

We analyzed CCE alkaline phosphatase (AP), Oct3/4 and nanog as markers of renewal. Reduction of the level of these factors is consistent with loss of renewal capacity. AP expression was assayed by staining. In brief, CCE colonies were rinsed with PBS 3 times, and fixed in cold 4% paraformaldehyde for 15 min at 4°C. After washing with PBS, they were incubated with Tris buffer containing 0.6 mg/ml Fast Red TR salt (Sigma) at room temperature for 1 h, washed with deionized water and examined under light microscope. Oct3/4 was analyzed by Western blotting and immunofluorescent staining. Nanog was analyzed by Western blotting.

### Assay of ROCK Activities

CCE cells were treated with 10 µM of Y-27632 or 10 µM of H-89 for 2 h, harvested and centrifuged. Cell pellets were resuspended in extraction buffer (50 mM Tris-HCl, pH 8.0, 0.1% triton X-100, 1 mM EDTA, 1 mM EGTA, 2 mM NaF, 2 mM Na_3_VO_4_, 10 mM beta-mercaptoethanol, protease inhibitor cocktail; Roche) and lysed by three cycles of freezing and thawing. Samples were centrifuged at 16,000×g for 15 min. Protein concentrations of supernatants were determined by Bio-Rad protein assay (Bio-Rad, USA). ROCK activity was determined using a Rho-kinase assay kit (Cyclex Co., Ltd., Japan) according to the manufacturer's protocol. This assay is based on precoating 96-well plates with a ROCK substrate, the C terminus of recombinant myosin-binding subunit (MBS) of myosine phosphatase. ROCKs phosphorylate Thr-696 of the substrate, and the phosphorylated MBS is detected with a horse radish peroxidase conjugated-phospho-MBS Thr-696 antibodies. The ROCK activity is measured by spectrophotometric analysis of color changes of tetramethylbenzidine (TMB). Recombinant ROCK II (Cyclex) was included as a positive control.

### Western Blot Analysis

Western blotting was performed as previously described [18]. In brief, CCE cells were washed and lysed in ice-cold RIPA buffer (Upstate, Lake Placid, NY) containing a protease inhibitor cocktail (Roche Diagnostics). 20–30 µg of lysate proteins were applied to each lane and separated on 8% SDS polyacrylmide gel eletrophoresis. The gels were transferred to PVDF membrane (Minipore Corp. Bedford, MA) and proteins were identified with specific murine antibodies and visualized by enhanced chemiluminescence (Pierce, Rockford, IL). Antibodies for identifying murine CCE proteins included (1) monoclonal antibodies against Flk-1 and Oct3/4, goat polyclonal antibodies against nestin, GATA-4 and actin and rabbit polyclonal antibodies against NG2 (all from Santa Cruz Biotechnology); (2) rabbit polyclonal antibodies against ROCK-1 and monoclonal antibodies against βIII-tubulin from Chemicon (Temecula, CA); (3) rabbit polyclonal antibodies against SOX-1 and monoclonal antibodies against MAP2c from Abcam (Cambridge, UK) and (4) rabbit polyclonal antibodies against ROCK-2 from Upstate.

### Immunofluorescence Staining

CCE cells were fixed with 4% paraformaldehyde for 10 min, washed, and permeabilized with methanol. After treatment with 3% BSA-0.1% Tween PBS for 30 min, they were incubated overnight at 4°C with Oct3/4 antibodies in 1% BSA-PBS, followed by incubation with FITC-conjugated secondary antibodies. The fluorescent image was detected with Olympus inverted confocal microscope.

### Quantitative mRNA Analysis

RNA was isolated by the Trizol RNA isolation system (Invitrogen). cDNA was synthesized from RNA with oligo-dT and SuperScript II reverse transcriptase (Invitrogen). cDNAs were used for PCR amplification with the forward primer, 5′-GAGAAGACAGTGAGGCAGATGAGTTA-3′ and the reverse primer, 5′-GCCTCTGTTCTCCAGCTTGCT-3′ for mouse nestin (113 bps) and 5′-ATCTACGAGGGCTATGCTCTCC-3′ and 5′-ACGCTCGGTCAGGATCTTCAT-3′ for actin (125 bps). Quantitative real-time PCR was performed according to the protocols provided by ABI PRISM 7900 system using 30 cycles of 95°C, 20 s; 60°C, 10 s; and 72°C, 10 s. The relative gene expression was obtained by ΔCT and ΔΔCT assay [ΔCT  =  CT _(Target gene)_ − CT _(β-actin)_ and ΔΔCT = ΔCT_(experimental group)_ −ΔCT_(control group)_]. All reactions were performed in triplicates and normalized by reference gene expression.

### Transfection of ROCK siRNA

40 nM of ROCK-1 or ROCK-2 siRNA, or a negative control RNA (B-Bridge International, Tokyo, Japan) was mixed with LipofectAMINE 2000 transfection reagent (Invitrogen) in opti-DMEM medium (Invitrogen) and added to CCE cells for 24 h. Cells were washed and replaced with fresh medium for an additional 24 h. The gene silencing effect was analyzed by Western blotting. The oligonucleotide sequences of ROCK-1 and ROCK-2 siRNA were ‘CCAAAUACCUCCUCAGUAATT’ and ‘GAAGAUAAAUCGUGCACUATT’, respectively.

### Statistical Analysis

ANOVA software was used to determine statistical differences. A p value <0.05 was considered to be statistically significant.

## Results

### Y-27632 or H-89 Inhibited ROCK Activities without Altering ROCK-1 or ROCK-2 Proteins in CCE Cells

**Figure 1:**
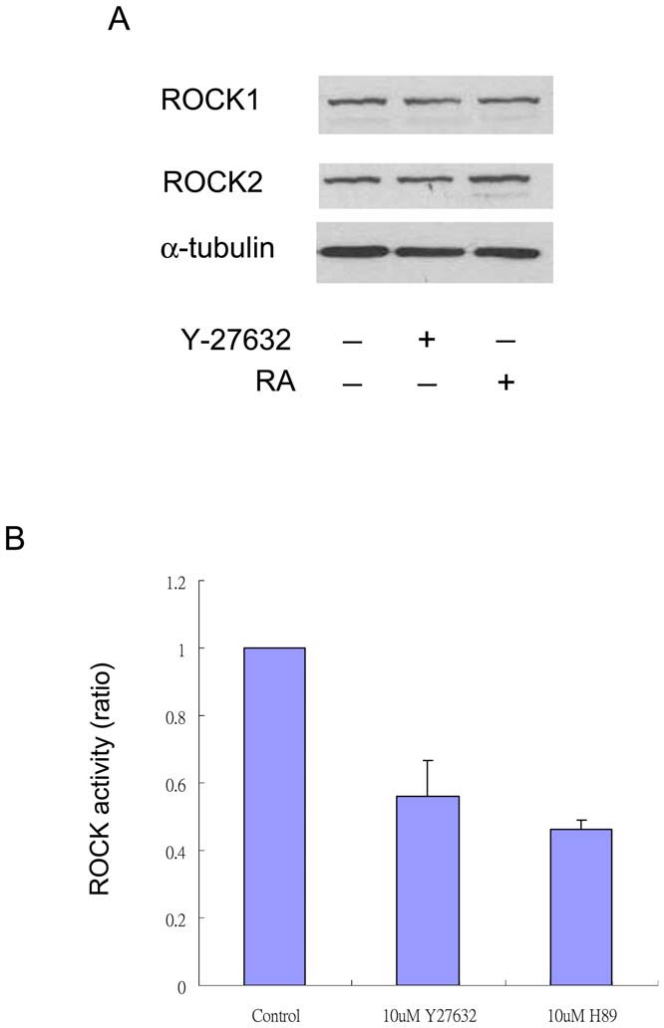
ROCK proteins and activities in CCE cells with & without Y-27632 treatment. ***A.*** CCE cells seeded at 10^4^ cells/cm^2^ for 24 h were treated with Y-27632 (10 µM) or RA (10 µM) for 48 h. ROCK-1 and ROCK-2 proteins were analyzed by Western blotting. ***B.*** ROCK activities in CCE cells treated with Y-27632 or H-89 (10 µM) for 2 h. Each bar denotes mean ± SEM of three experiments.

ROCK-1 and ROCK-2 proteins and ROCK activities were detected in CCE cells (Fig. 1A and 1B). Y-27632 (10 µM) inhibited ROCK activities by ∼50% (Fig. 1B) without altering ROCK protein levels (Fig. 1A). H-89 at 10 µM inhibited ROCK activity to an extent similar to Y-27632 (Fig. 1B).

### ROCK inhibitors Prevented and Disrupted CCE Colony Formation

**Figure 2:**
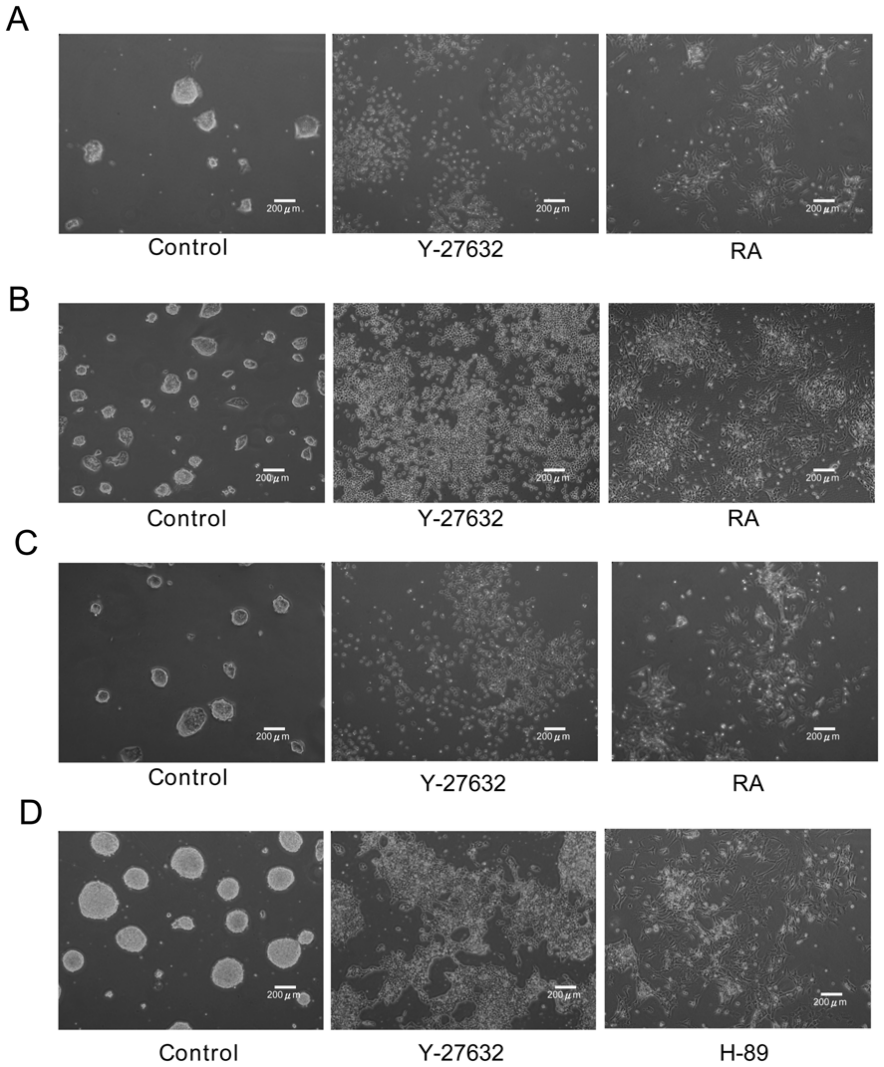
Effect of Y-27632 on CCE colony formation and morphological changes. ***A & B.*** Cells in suspension were treated with Y-27632 or RA and seeded at 2×10^3^ cells/cm^2^ for 96 h (**A**) or 1×10^4^ cells/cm^2^ for 48 h (***B***). ***C.*** Cell suspensions were seeded at 2×10^3^ cells/cm^2^ for 24 h followed by treatment with the indicated agent for 96 h. ***D.*** Cell suspensions seeded at 1×10^4^ cells/cm^2^ for 24 h were treated with the indicated agent for 48 h. Cells were examined under phase microscopy.

CCE cell suspensions harvested by trypsin were treated with Y-27632 and seeded at a low cell density (2×10^3^ cells/cm^2^) or at a high density (1×10^4^ cells/cm^2^) for 48–96 h. Colony formation was examined. The control cells formed discrete colonies while cells treated with Y-27632 did not form colonies and grew as individual cells regardless of the initial seeding density (Fig. 2A and 2B). To determine whether ROCK inhibitors disrupt preformed colonies, we seeded CCE cells at either density for 24 h to allow for colony formation and added Y-27632 to the cultured dish. CCE colonies were detected after 24 h seeding which increased in numbers and sizes over 48–96 h (Fig. 2C and 2D, control). Y-27632 treatment resulted in complete disruption of colonies at low or high seeding density and cells grew as individual cells (Fig. 2C and 2D). H-89 treatment also disrupted colonies and induced cells to grow as monolayers (Fig. 2D). Treatment with retinoic acid (RA) prevented colony formation and induced collapse of preformed colonies as Y-27632 but RA treated cells underwent cell death and therefore were lower in numbers than Y-27632-treated cells (Fig. 2A-C).

### Y-27632 Altered CCE Renewal Properties in a Cell-Density Dependent Manner

**Figure 3:**
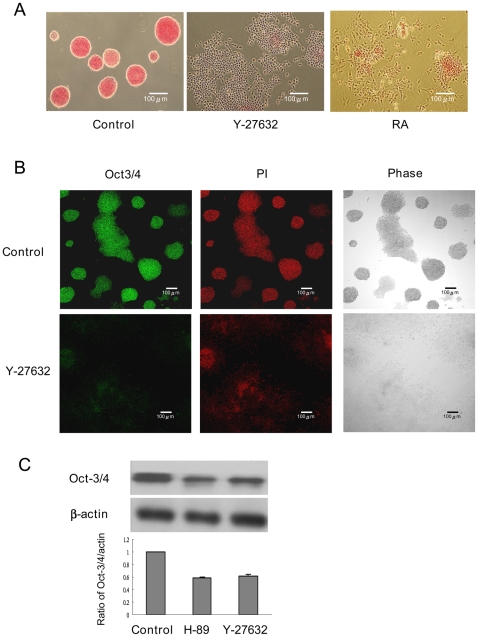
Reduction of AP and Oct3/4 by ROCK inhibition. CCE cells seeded at 1×10^4^ cells/cm^2^ for 24 h were treated with Y-27632 or other agents for 48 h and cells were analyzed with AP staining (***A***), Oct3/4 immunofluorescence (***B***) and Oct3/4 Western blotting (***C***). The blots in Figure 3C were quantitated by densitometry. Each bar denotes mean ± SEM of three experiments. PI denotes propidium iodide staining and phase, phase contrast microscopy.

**Figure 4:**
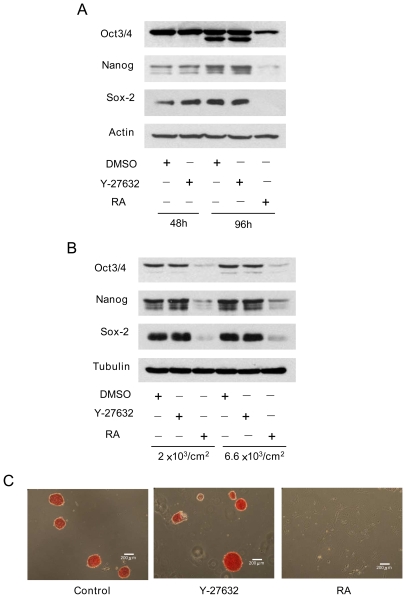
Retaining of renewal properties at low seeding density. ***A.*** Western blot analysis of renewal factors in CCE cells seeded at low-density (2×10^3^ cells/cm^2^) for 24 h followed by treatment with Y-27632 (10 µM), RA (10 µM) or DMSO vehicle for 48 h and 96 h. ***B.*** Western blot analysis of renewal factors in CCE cells seeded at 6.6×10^3^ vs. 2×10^3^ cells/cm^2^ for 24 h and treated with inhibitors for 96 h. ***C*** **.** Low-density CCE cells were treated with vehicle control or Y-27632 for 96 h, harvested and reseeded at 2×10^3^ cells/cm^2^ for 48 h. Cells were stained for AP.

Y-27632-treated CCE cells seeded at high density (1×10^4^ cells/cm^2^) lost AP staining of individual cells (Fig 3A) and had reduced Oct3/4 protein expressions as analyzed by immunofluorescence (Fig. 3B) and Western blotting (Fig. 3C). H-89 also reduced Oct3/4 (Fig. 3C). By contrast, Y-27632-treated cells seeded at low density (2×10^3^ cells/cm^2^) retained Oct3/4, nanog and SOX-2 proteins (Fig. 4A) and did not lose renewal factors even at a seeding density of 6.6×10^3^ cells/cm^2^ (Fig. 4B). Oct3/4 proteins were detected as a single band in cells treated with Y-27632 or DMSO control for 48 h while an additional fast-moving band was detected in cells treated with Y-27632 or DMSO for 96 h. Nanog proteins were detected by Western blotting as double bands. The reason for the double bands is unclear but may be explained by post-translational modification such as phosphorylation. To ascertain that Y-27632-treated individual cells retain ES properties, we washed and trypsinized them, and re-seeded them for 96 h. The re-seeded cells formed AP-positive colonies while RA-treated CCE cells failed to form colonies or AP-positive cells after reseeding (Fig. 4C).

### Y-27632 Induced CCE Differentiation into Neural Progenitor Cells (NPCs) at High Seeding Density

**Figure 5:**
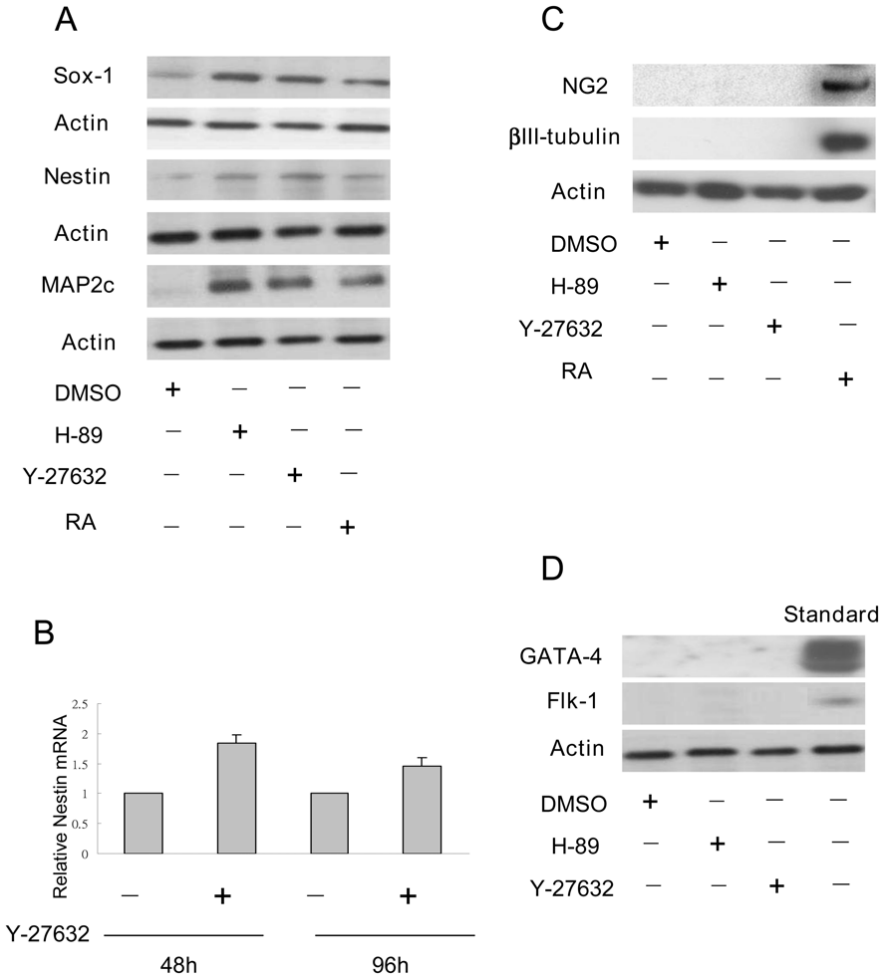
Expression of NPC markers in high-density CCE cells treated with ROCK inhibitors. CCE cells seeded at 1×10^4^ cells/cm^2^ for 24 h were treated with the indicated inhibitors for 48 h. ***A***. Nestin and other NPC proteins were analyzed by Western blotting. ***B***. Nestin mRNA levels were analyzed by quantitative PCR. Each bar denotes mean ± SEM of three experiments. ***C***. Neuronal markers and ***D***. GATA-4 and FIK-1 proteins were analyzed by Western blotting. The blots are representative of those from three experiments.

As Y-27632-treated CCE cells seeded at 10^4^/cm^2^ lost renewal properties, we determined if they express differentiation markers. Y-27632 and H-89 treated cells expressed an increased level of SOX-1, nestin and MAP2c proteins compared to DMSO control (Fig. 5A). Y-27632 increased nestin mRNA expression at 48 h and 96 h (Fig. 5B), suggesting that nestin regulation by Y-27632 occurs at the transcriptional level. Neither Y-27632 nor H-89 induced the expression of neuronal markers such as NG-2 and β-III tubulin (Fig. 5C) or endoderm and mesoderm markers such as GATA-4 and Flk-1 (Fig. 5D). As expected, RA treatment resulted in increased expression of markers of NPCs and mature neurons (Fig. 5A and 5C). CCE cells seeded at 2×10^3^ cells/cm^2^ did not express differentiation markers after treatment with Y-27632 for 48 h and 96 h (data not shown). These results indicate that the fate of Y-27632-treated CCE cells depends on seeding cell density. It is at high seeding density that Y-27632 causes individually growing cells to lose renewal factors and differentiate selectively into NPC lineage.

### ROCK-1 and ROCK-2 siRNA Independently Induced CCE Differentiation into NPCs

**Figure 6:**
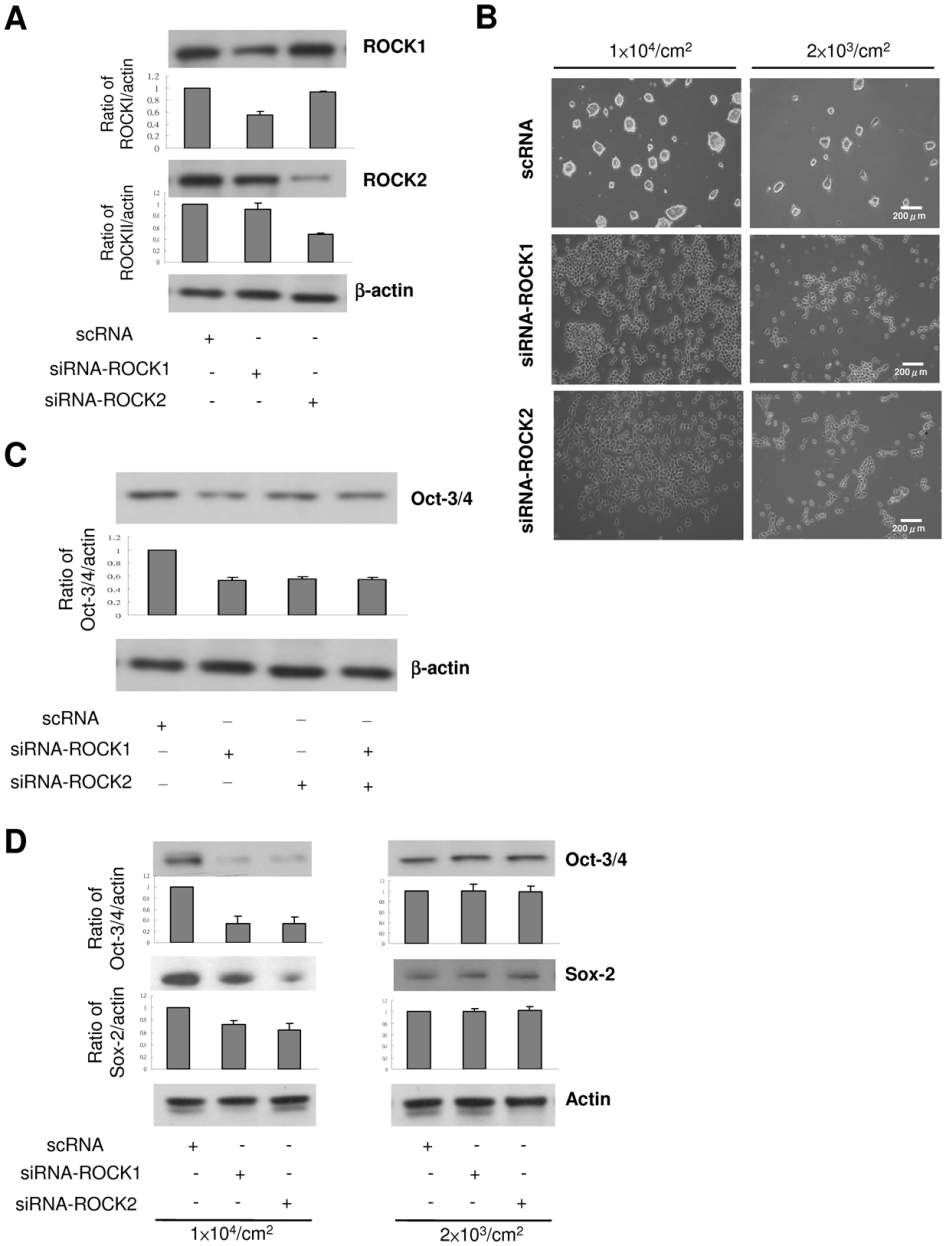
Influence of ROCK siRNA on colony formation and renewal factors. ***A***. CCE cells transfected with selective ROCK-1 and ROCK-2 siRNA or control RNA were seeded at 10^4^ cells/cm^2^ for 48 h, and ROCK-1 and ROCK-2 proteins were analyzed by Western blotting. The blots were quantitated by densitometry. Each bar denotes mean ± SEM (n = 3). ***B.*** Colony formation and growing pattern were examined under phase-contrast microscopy. ***C***. Cells at 1×10^4^ cells/cm^2^ were treated with ROCK-1 plus ROCK-2 siRNA vs. individual ROCK siRNA. Oct3/4 proteins were analyzed by Western blotting. ***D.*** Oct3/4 and SOX-2 proteins were analyzed in siRNA-treated cells seeded at 1×10^4^ cells/cm^2^ (left panel) vs. 2×10^3^ cells/cm^2^ (right panel). The blots were analyzed by densitometry. Each bar denotes mean ± SEM (n = 3).

**Figure 7:**
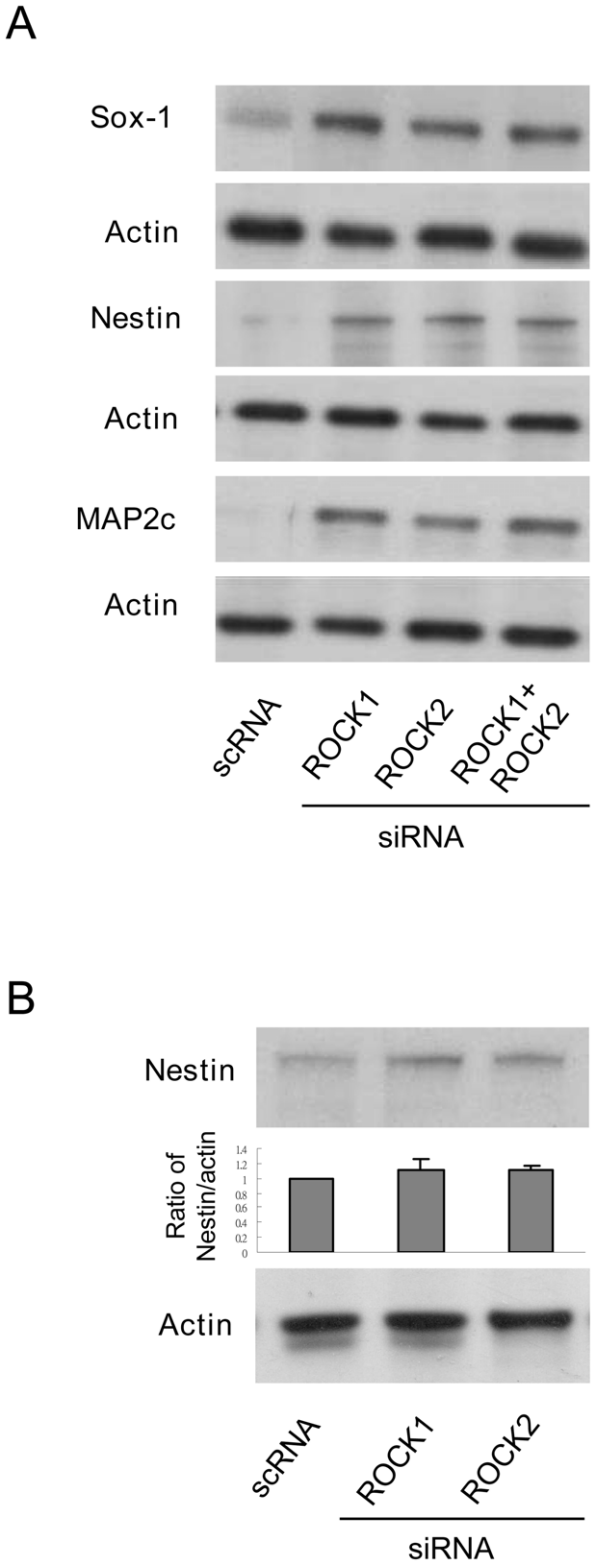
Regulation of NPC markers by ROCK siRNA in a cell density-dependent manner. ***A***. CCE cells were seeded at 1×10^4^ cells/cm^2^ for 48 h and SOX-1, nestin and MAP2c proteins in CCE cell lysates were analyzed by Western blotting. ***B***. CCE cells were seeded at 2×10^3^ cells/cm^2^ for 48 h and nestin proteins were analyzed by Western blotting. The blot was quantitated with densitometry. Each bar denotes mean ± SEM (n = 3).

To provide direct evidence for involvement of ROCK in ES cell differentiation, we transfected CCE cells with ROCK-1 or ROCK-2 siRNA or control RNA. The transfected cells were seeded at 10^4^ cells/cm^2^. ROCK-1 siRNA suppressed ROCK-1 proteins by ∼50% while ROCK-2 siRNA selectively suppressed ROCK-2 by ∼70% (Fig. 6A). ROCK-1 or ROCK-2 siRNA individually disrupted CCE colony formation and induced CCE to grow as individual cells with morphological changes similar to Y-27632 treatment (Fig. 6B). ROCK-1 and ROCK-2 siRNA caused a reduction of Oct3/4 proteins to a similar extent (Fig. 6C). Treatment of CCE with combined ROCK-1 and ROCK-2 siRNA reduced Oct3/4 to an extent comparable to treatment with ROCK-1 or ROCK-2 alone (Fig. 6C). By contrast, neither ROCK-1 nor ROCK-2 siRNA reduced Oct3/4 or SOX-2 proteins in cells at seeding density of 2×10^3^ cells/cm^2^ as compared to those at seeding density of 1×10^4^ cells/cm^2^ (Fig. 6D). ROCK-1 or ROCK-2 siRNA individually induced SOX-1, nestin and MAP2c proteins in cells seeded at 1×10^4^ cells/cm^2^, and combined ROCK-1 and ROCK-2 siRNA did not have an additive or synergistic effect (Fig. 7A). In contrast, neither ROCK-1 nor ROCK-2 siRNA significantly altered nestin protein levels in cells seeded at 2×10^3^ cells/cm^2^ (Fig. 7B). These results suggest that ROCK-1 and ROCK-2 control murine ES cell renewal and NPC differentiation fate by independent actions which do not overlap and cannot mutually compensate.

## Discussion

Our results show that ROCK-1 and ROCK-2 are constitutively expressed and active in CCE murine ES cells under normal culture conditions. By using pharmacological inhibitors and genetic silencers of ROCK-1 and ROCK-2, our data provide evidence for a critical role that both ROCKs play in promoting ES cell growth as colonies and maintaining ES cell at undifferentiated state. Once ROCK activities are inhibited or ROCK protein expressions are suppressed, CCE cells are no longer able to grow into colonies but grow as individual cells. It was thought that when ES colonies destabilize and spread out as individual cells, they have lost the renewal properties and have undergone differentiation. However, a recent report has shown that monolayer ES cells still retain undifferentiated state and was able to grow as colonies when reseeded [19]. Human ES cell colonies dissociated by Y-27632 retain renewal properties and are resistant to apoptosis [20]. We confirm in this study that CCE cells seeded at low densities grow as individual cells after Y-27632 treatment. They retain renewal factors and do not express differentiation markers. Furthermore, they are capable of growing as colonies after removal of Y-27632 and re-seeding. We provide novel information in this study that CCE cells seeded at a high density, i.e., 10^4^ cells/cm^2^ lose renewal properties and differentiate into cells of NPC lineage after ROCK inhibition with Y-27632 or RNAi. These cells appear to have irreversibly lost the renewal properties. Taken together, these findings indicate that ROCKs play multiple roles in regulating ES cell renewal. They are required for ES cells to grow as colonies regardless of the seeding density and are essential for maintaining ES cells in the undifferentiated state only at high seeding cell density.

One possible reason for involvement of ROCKs in maintaining murine ES cells at the undifferentiated state only at high seeding densities is activation of ROCK signaling by cell-cell contact. CCE cells seeded at 10^4^ cells/cm^2^ occupy about 50–70% of culture dish surface and have ample opportunities for cell-cell contact, while CCE cells seeded at 2×10^3^ cells/cm^2^ spread on culture dish sparsely with little cell-cell contact. Cell-cell contact may trigger intracellular signaling pathways such as the β-catenin pathway [21]. A pool of β-catenin is localized to plasma membrane where it is associated with E-cadherin, bridges E-cadherin with cytoskeleton and regulates cell-cell interaction [22]. Another pool of β-catenin is localized to cytoplasm where it is associated with APC-axin-GSK-3β complex. At resting cellular state, it is phosphorylated by GSK-3β and degraded via ubiquitin-proteasome [23], [24]. Upon Wnt activation, GSK-3β is inhibited and free cytosolic β-catenin is translocated to nucleus where it interacts with Tcf/Lef transcriptional factor and activates Tcf/Lef-mediated gene expression [25]. The subcellular pools of β-catenin are regulated by cell density. It was reported that β-catenin in human keratinocytes is diffusely distributed at low cell densities and is predominantly located to plasma membrane after cells have reached confluency [26]. It was further reported in murine ES cells that at high seeding density, β-catenin is predominantly localized to plasma membrane which is associated with reduction of Tcf/Lef transcriptional activation and suppression of neural differentiation induced by retinoic acid [27]. Results from these reports suggest a dynamic regulation of β-catenin subcellualr localization by cell densities in culture. As CCE cells at high but not low seeding density lose the renewal properties and differentiate into NPCs, it may be speculated that ROCK inhibition brings about dys-regulation of β-catenin localization induced by cell-cell contact and thereby changes β-catenin-mediated murine ES cell renewal and differentiation [28]. Work is in progress to test this hypothesis.

It is unclear why ROCK inhibition at high cell density induces selective CCE cell differentiation into SOX-1^+^, nestin^+^ and MAP2c^+^ NPCs in the absence of defined neural differentiation media. One possible explanation is that once the ES cell renewal program is disrupted by ROCK inhibition, cells undergo neural differentiation by default [29], [30]. It is also possible that ROCKs may play a dual role in promoting renewal and blocking neural differentiation. When ROCKs are suppressed in CCE cells, renewal properties are lost and neural differentiation control is disrupted, leading to generation of NPCs.

Our results reveal a new approach for preparing NPCs for therapeutic use. About 20–30% of CCE cells seeded at high density express NPC markers after treatment with Y-27632 for 48 h. With sorting of NPC positive cells, it is possible to obtain a large number of NPCs (SOX-1^+^, nestin^+^ and MAP2c^+^). To have clinical relevance, it will be necessary to ascertain that ROCK inhibition of human ES cells seeded at high density yields NPCs. When confirmed, this method of preparing NPCs from ES cells should have major therapeutic use in nerve repair and treatment of diverse neurological diseases.
