# Correction: Rho Kinases Regulate the Renewal and Neural Differentiation of Embryonic Stem Cells in a Cell Plating Density–Dependent Manner

**DOI:** 10.1371/annotation/cf10edef-3ca9-413f-9c97-98b628c29a25

**Published:** 2010-03-17

**Authors:** Tzu-Ching Chang, Yen-Chung Chen, Ming-Hua Yang, Chien-Hung Chen, En-Wei Hsing, Bor-Sheng Ko, Jun-Yang Liou, Kenneth K. Wu

A contribution was omitted. Jun-Yang Liou's contribution includes "Financial and administrative support". 

