# Correction: Rho Kinases Regulate the Renewal and Neural Differentiation of Embryonic Stem Cells in a Cell Plating Density–Dependent Manner

**DOI:** 10.1371/annotation/3490bca9-a5da-46c4-a317-de840b7f23f2

**Published:** 2010-04-09

**Authors:** Tzu-Ching Chang, Yen-Chung Chen, Ming-Hua Yang, Chien-Hung Chen, En-Wei Hsing, Bor-Sheng Ko, Jun-Yang Liou, Kenneth K. Wu

The seventh author of this paper, Dr. Jun-Yang Liou, is a co-corresponding author of this paper with Kenneth K. Wu. His e-mail address is jliou@nhri.org.tw.

